# The Basis and Applications of the Action Fluency and Action Naming Tasks

**DOI:** 10.1590/S1980-57642014DN81000008

**Published:** 2014

**Authors:** Bárbara Costa Beber, Márcia L.F. Chaves

**Affiliations:** 1 Dementia Clinic, Neurology Service, Hospital de Clínicas de Porto Alegre (HCPA), RS, Brazil.; 2 Programa de Pós-Graduação em Ciências Médicas da Faculdade de Medicina, Universidade Federal do Rio Grande do Sul (UFRGS), RS, Brazil.; 3 PhD Schoolarship, CAPES.; 4 Departamento de Medicina Interna da Faculdade de Medicina, UFRGS, Porto Alegre, RS, Brazil.

**Keywords:** language, cognition, neuropsychological tests, neuropsychology

## Abstract

The Action Fluency (AF) and Action Naming (AN) are different tasks involving verb generation. Evidence indicates that verb tasks yield different information from that obtained with nouns.

**Objective:**

This study aimed to analyze the information available in the scientific literature on the mechanism and clinical application of these tasks.

**Methods:**

We carried out a systematic review of the literature and the findings were presented according to clinical studies and neuroimaging studies, and to the task in question.

**Results:**

The literature contained a variety of relevant studies with different objectives, methodologies and populations. After the analysis (exclusion criteria) of the studies obtained by the search terms, only 40 studies were included in this review.

**Conclusion:**

It was possible to conclude that AF and AN involve different brain processes, and although recruiting frontal areas and circuits, other areas are also critical. These tasks may be useful for differentiating Primary Progressive Aphasias; AF might represent a new measure of executive function; finally, both these tests can be used to provide a better understanding of cognitive processes and certain diseases.

## Abstract (pt)

A Fluência de Ações (FA) e a Nomeação de Ações (NA) são diferentes tarefas que exigem geração de verbos. Evidências indicam que tarefas que utilizam verbos fornecem informações diferentes daquelas que utilizam substantivos.

**Objetivo:**

Este estudo objetivou analisar a informação disponível na literatura científica sobre mecanismos e aplicações clínicas destas tarefas.

**Métodos:**

Foi realizada uma revisão de literatura sistemática e os achados foram apresentados de acordo com estudos clínicos e de neuroimagem, e de acordo com o tipo de tarefa em questão.

**Resultados:**

A literatura apresentou estudos diversificados sobre o assunto, com diferentes objetivos, metodologias e populações. Após análise dos dados (com critérios de exclusão) dos estudos obtidos na busca, apenas 40 estudos foram incluídos nesta revisão.

**Conclusão:**

Foi possível concluir que FA e NA envolvem diferentes áreas cerebrais, embora envolvam áreas e circuitos frontais, outras áreas também são críticas. Tais tarefas podem ser importantes na diferenciação das Afasias Progressivas Primárias; a FA pode ser uma nova medida de funções executivas; e, finalmente, ambos podem ser usados para permitir um melhor entendimento de processos cognitivos e de determinadas doenças.

## INTRODUCTION

The need to improve the diagnosis of neurological disorders that affect cognition prompts the development of specific neuropsychological evaluations - practical and efficient - both in clinical practice and in research. The motivation to study the brain's ability to name or to spontaneously produce words (fluency), as well as to better comprehend its neural basis, emerged from the evolution of the investigation of language and memory. At first, the main focus was the understanding of nouns - in general, the name of things - and later of verbs - that is, the name of actions.

In fact, some studies on neuroimaging and generation of verbs and nouns have shown that verbs and nouns can activate different brain areas.^1-4^ Past and recent studies in patients with various kinds of brain impairment have reported different performance between verbs and nouns according to the brain area affected.^5-11^ These findings suggest that the testing of verb generation can have clinical and scientific relevance for the understanding of neurobiology of language and brain diseases. The most applied and well known tasks on verb generation are the fluency and naming tasks. Evidence indicates that verb fluency or naming tasks provide different information from that obtained with nouns. However, questions remain regarding the state of the art of the mechanisms and clinical applications of fluency and/or verb naming tasks.

The Action Fluency (AF) task is defined as a measure of the ability to generate verbs in a period of time (1 minute)^12^ whereas the Action Naming (AN) task is defined as the ability to name a limited amount of verbs graphically represented.^13,14^ Different ways of applying the AN have been described in the literature, demonstrating the need to better understand the task for further standardization. These two tasks are used to verify the ability to generate and evoke verbs (or actions), but the AN task more frequently appears in the studies (Table 1).

**Table 1:** Included and excluded studies.

Searched Expressions	Total published in last 15 years	Excluded: other tasks or goals	Excluded: literature review or case report	Excluded: other population	Excluded: other language	Excluded: repeated in previous search	Not accessible	Total included
Verb fluency AND cerebral cortex/physiopathology	8	2	1	1	1	0	0	3
Action fluency AND cerebral cortex/physiopathology	4	1	1	0	0	1	0	1
Action naming AND cerebral cortex/physiopathology	20	9	5	1	0	1	0	4
Verb naming AND cerebral cortex/physiopathology	15	9	2	0	0	4	0	0
Action naming AND brain mapping/methods	3	2	0	0	0	0	0	1
Verb naming AND brain mapping/methods	5	2	0	0	0	1	1	1
Verb fluency AND brain mapping/methods	5	4	0	0	0	1	0	0
Action fluency AND brain mapping/methods	2	2	0	0	0	0	0	0
Verb naming AND neuropsychological tests	38	14	7	0	0	5	2	10
Action naming AND neuropsychological tests	65	31	11	1	1	12	3	6
Action fluency AND neuropsychological tests	44	15	5	5	2	6	0	11
Verb fluency AND neuropsychological tests	26	9	0	4	1	9	0	3
Total	235	100	32	12	5	40	6	40

Many studies have used these two tasks interchangeably as if both were able to provide data on verb recall. However, it is important to raise the following question: if AF requires generation of verbs without visual cues, and AN requires naming for a visual stimulus, do these two tasks provide data on the same cognitive process? In fact, what do these tasks represent?

To better understand the meaning of each task it is necessary to appreciate the scientific evidence on cognitive processes involved in fluency and verb naming. Therefore, the objective of this study was to analyze the information available in the scientific literature about the mechanism (brain processes) and clinical applications of the AF and AN tasks. For this purpose we carried out a systematic review of the literature on verb generation from neurological, psychological and/or linguistic perspectives.

## METHODS

The PUBMED database was used to perform the systematic review with the terms displayed in Table 1. The terms used for the systematic review were searched for all fields of a published paper. Inclusions were original English articles, published in the last 15 years (1999-2013), involving adults and/or elderly participants, and studies that used AF and/or AN. Exclusions were literature reviews and case reports, and those studies employing different stimuli or objectives, such as no pictures for the AN.

Articles that appeared repeatedly among searches were only included on the first instance and subsequent repetitions excluded. The search was carried out in December 2013 and a total of 40 studies were selected.

## RESULTS

### Clinical studies with Action Fluency

*Action Fluency in healthy subjects* - Assessing how AF is processed in normal subjects is important, as is obtaining normative data, for a better understanding of this function. In the present review, we found four studies with AF normative data, one with American English speaking young adults,^15^ another with American English speaking elderly individuals,^16^ one study with normative data in Spanish young adults,^17^ and one normative study in elderly Danish subjects.^18^ We found one study that showed and compared data on healthy adults and healthy elderly individuals. Two investigations with validity information on AF were retrieved.^12,15^

In the study with young adults (n=174), a series of analyses had been conducted to examine the psychometric properties of AF. The first set of analyses described the development of demographically adjusted AF normative data. Subsequently, a group of hypothesis-driven correlational analyses revealed significant associations between AF and putative tests of executive functions, verbal working memory, verbal fluency and information processing speed, but not between AF and tests of learning or constructional praxis. The final set of analyses demonstrated the test-retest stability of the AF test and provided standards for determining statistically reliable changes in performance.^15^ The variable found to most influence AF is education.^16-18^

AF construct validity was carried out in a group of healthy elderly control subjects (n=67).^12^ Modest to moderate relationships between AF and several executive measures were observed. No correlation between AF and semantic and episodic memory was observed, however, education correlated with AF. The authors supported the construct validity of AF as an executive function measure. The education adjusted AF performance of healthy adults (n=36) was compared to that of healthy elderly individuals (n=30) in other study.^19^ Elderly individuals presented lower AF scores.

*Action Fluency in Neurodegenerative Disorders* - Action Fluency compared to noun and letter-based fluency was analyzed in subjects with Subjective Cognitive Impairment (SCI) (n=40), Mild Cognitive Impairment (MCI) (n=60), and Alzheimer's Disease (AD) (n=57).^9^ AF performance differed significantly between SCI and AD groups, where AD patients exhibited lower scores on this task. Another investigation evaluated verbal fluency performance (category, letter and action fluency) in MCI (n=37), SCI (n=37) and a group of healthy controls (n=33).^10^ The investigation found no significant differences for AF between these groups.

The AF test was tested to verify whether it has value for the differential diagnosis of AD and dementia with Lewy bodies (DLB). The study assessed animal fluency and AF, but only the AF differed significantly between the two demented groups, with DLB patients performing worse than AD patients.^20^

A study with normal pressure hydrocephalus (NPH), behavioral variant Frontotemporal Dementia (bv-FTD), nonfluent Primary Progressive Aphasia (PPA), and AD patients was carried out to test the following hypotheses:

a) subcortical dementia and dementias predominantly affecting frontal cortex present more difficulty on AF than noun fluency tasks;
b) AD, with temporo-parietal cortical dysfunction, is associated with greater difficulty in noun than verb fluency.^21^

A total of 234 participants, including healthy controls (n=20), NPH (n=144), AD (n=33), bv-FTD (n=22) or nonfluent PPA (n=15), were included in the investigation. Tests employed were animal fluency, AF, letter fluency, and the Mini-Mental State Examination (to control for severity of dementia). The NPH and bv-FTD/PNFA patients had significantly higher MMSE and animal fluency scores, and lower AF than AD patients (age adjusted). Only NPH and bvFTD/nonfluent PPA patients showed significantly lower scores on AF than animal fluency. The authors concluded that the findings confirmed their hypotheses.

In Parkinson's disease (PD), lexical, semantic, and AF were evaluated by comparing PD patients with and without dementia against elderly controls. The results showed an interaction between fluency type and subject group. PD with dementia performed significantly more poorly than their non-demented counterparts and normal controls on all three fluency tasks, but a prominent disparity in scores was noted on the AF task.^7^

A follow-up study of 24 months with PD patients (n=20) and healthy controls (n=20) matched for age, education and gender was carried out.^22^ The results showed that AF was significantly lower in PD patients since baseline and remained stable throughout the follow-up.

In a comparison of letter, category, and AF tests among healthy younger and older adults, PD patients (without dementia), and AD patients, PD patients showed poorer scores on the AF task.^19^

An evaluation of Friedreich's ataxia patients showed significantly higher impairment in letter fluency and AF compared to a group of healthy controls. Based on the analysis, the authors concluded this deficit did not occur as a consequence of dysarthria or cognitive slowing, but due to impaired executive control (a dysfunction of cerebellum-prefrontal cortex connections).^23^

*Action Fluency in schizophrenia* - Badcock and co-workers hypothesized that impaired production of action-related language might be an important mechanism supporting thought disorder schizophrenia.^24^ To test their hypothesis, semantic, phonological, and action fluencies were tested in schizophrenia patients (n=53) and healthy controls (n=69). The authors also used the Schizotypal Personality Questionnaire (SPQ) to index odd and disorganized speech, as well as positive and negative symptoms. Schizophrenia patients showed impairment on all fluency tasks. Among the schizophrenia patients, Odd Speech was correlated with poor fluency for actions, tools and musical instruments but not for fruit or phonological fluency. These findings pointed to a single connection and common etiology between AF and odd speech in schizophrenia rather than a general impairment in language/executive functions.

The study of Woods and associates also found greater impairment in all fluencies among schizophrenia patients (n=22) compared with matched controls (n=27). However, only AF showed significant correlations with working memory, response inhibition, semantic memory and cognitive flexibility.^25^

*Action Fluency in HIV* - Studies in patients with HIV-1 have also contributed to understanding on AF. The HIV-1 infection promotes an inflammatory response in the central nervous system that is characterized by viral replication in perivascular macrophages, chronic activation of microglia, and accumulation of neurotoxic cellular byproducts (Gonzalez-Scarano & Martín-García, 2004 cited by Woods et al., 2006).^26^ Although HIV-1 does not infect neurons, wide-spread neuronal and glial pathology (such as leukoencephalopathy) is frequent, particularly affecting the basal ganglia and the fronto-striato-thalamocortical circuits (Langford & Masliah Everall 2005 cited by Woods et al., 2006).^26^ Proportionally to frontostriatal neuropathogenesis, the neuropsychological sequelae of HIV-1 include bradyphrenia, bradykinesia, executive dysfunction, impaired encoding and recall of episodic memory.^26^ Action fluency showed poorer performance than noun fluency (semantic memory) in patients infected with HIV-1 (n=97).^27^

Based on the hypothesis that AF is more sensitive to neuropsychological impairments in HIV-1 than other fluency tasks, and reflects inefficiencies in the motor representation necessary to carry out schemes of action of instrumental activities of daily living, the ecological validity of AF was evaluated as a predictor of instrumental activities of daily living (IADL) among patients with HIV-1. Action, letter (FAS), and noun (animal) fluencies were compared in HIV-1-infected patients (n=21) with self-reported IADL dependence relative to demographically comparable HIV-1-infected participants (n=76) who reported no IADL decline. Significant differences in action and letter fluency, but not in noun fluency, were observed between groups. Action fluency was more sensitive than letter fluency in classifying IADL dependent participants, and accuracy attained 76% (overall hit rate).^26^ Other investigations have shown a correlation between biomarkers and both noun and AF performance in HIV-1 patients (n=74). Deficits of AF were significantly associated with higher levels of S100β (a marker of astrocyte activation) in cerebrospinal fluid (CSF). S100β was also associated with executive function, but not with semantic memory or psychomotor speed.^28^

The main results for clinical studies using AF are summarized in Table 2.

**Table 2:** Summary of main results in clinical studies with AF.

Studies	Clinical Groups Studied	What was analyzed about Fluency?	Main Results
Ostberg et al. (2005)	SCI, MCI, AD	Category fluency, letter fluency, AF	AF: AD<SCI
Nutter-Upham et al. (2008)	MCI, SCI, HC	Category fluency, letter fluency, AF	AF: no significant differences between groups
Delbeuck et al. (2013)	AD, DLB	Category fluency, AF	AF: DLB<AD
Davis et al. (2010)	NPH, bvFTD, nfPPA, AD	Category fluency, letter fluency, AF	AF: NPH, bvFTD, nfPPA<ADNPH, bvFTD, nfPPA: AF<category fluency
Piatt et al. (1999)	PD with dementia, PD without dementia	Category fluency, letter fluency, AF	AF: PD with dementia<PD without dementia
Signorini & Volpato (2006)	PD, HC	Category fluency, letter fluency, AF	AF: PD<HC
McDowd et al. (2011)	Young adults, healthy older adults, AD, PD (without dementia)	Category fluency, letter fluency, AF	AF: PD<all other groups
De Nóbrega et al. (2007)	FA, HC	Category fluency, letter fluency, AF	AF: FA<HC
Badcock et al. (2011), Woods et al. (2007)	Schizophrenia, HC	Category fluency, letter fluency, AF	AF: Schizophrenia<HC
Woods et al. (2006)	HIV-1 IADL dependent, HIV-1 IADL independent	Category fluency, letter fluency, AF	AF: HIV-1 IADL dependent< HIV-1 IADL independent
Woods et al. (2005)	HIV-1	Category fluency, AF	AF<category fluency

AD: Alzheimer disease; bvFTD: behavioral variant Frontotemporal Dementia; DLB: dementia with Lewy bodies; FA: Friedreich ataxia; HC: healthy control; IADL: instrumental activities of daily living; MCI: Mild Cognitive Impairment; nfPPA : nonfluent Primary Progressive Aphasia; NPH: normal pressure hydrocephalus; PD: Parkinson disease; SCI: Subjective Cognitive Impairment.

### Clinical Studies with Action Naming

*Action Naming in healthy subjects* - According to a previous assumption that AN was impaired with normal aging, a study with healthy adults (n=66) aged between 30 and 79 years compared three AN assessments over a period of seven years.^29^ Performances of AN significantly declined on the second and third assessments for all participants older than 50 years at baseline.

*Action Naming in Neurodegenerative Disorders* - A group of investigators examined the differences between ON and AN tasks in patients with mild to moderate AD (n=19) in comparison to age-matched healthy subjects (n=19).^13^ They used a set of stimuli with object and action-matched items and collected not only accuracy data but also naming latencies. Both patients and healthy participants responded faster and made fewer errors on the object pictures than the action pictures. A further analysis of types of naming errors showed that ON and AN were associated with different types of errors. Action Naming was more problematic than ON for both patients and the healthy group, but the number of omission errors was equal in AN and ON for patients but negligible for healthy controls. However, ON elicited more visual and coordinate errors than AN for all participants. These findings showed that naming actions were not more difficult than naming objects, but had different demands.

A group of patients with MCI due to hippocampal atrophy (n=36) and a group of MCI due to multiple subcortical infarcts (n=41) had their psychological patterns investigated in order to assess whether they were showing typical markers of AD or vascular dementia (VD).^11^ The group of MCI due to multiple subcortical infarcts showed significant impairment on action naming compared with the group of MCI due to hippocampal atrophy and controls, while ON was impaired in both MCI groups.

In a study with different FTD variants, nonfluent PPA (n=15) and Amyotrophic Lateral Sclerosis (ALS) with FTD (FTD-ALS) (n=6) showed more impairment on verb naming than on noun naming.^8^ These patients also showed greater oral than writing difficulties when carrying out the naming tasks. Patients with fluent PPA showed the opposite performance pattern (more noun naming impairment and greater writing difficulty).

Similar findings were obtained in other evaluations of cases of bvFTD (n=16), nonfluent PPA (n=2), semantic dementia (SD) (n=6), progressive supranuclear palsy (PSP) (n=10), corticobasal degeneration (CBD) (n=10), AD (n=10) and controls (n=10).^30^ All groups of patients except for semantic dementia, presented greater AN impairment than ON. The discrepancy was similar in both bvFTD and AD groups, while nonfluent PPA, PSP and CBD groups had worst action than noun production.

To explore the contribution of the motor system for the representation of verbs, the ability of PD patients without dementia (n=28) to name matched sets of object and action pictures was evaluated. PD patients were compared to a group of healthy seniors (n=28), and a group of AD patients (n=28). PD patients presented significant impairment in their capacity to name actions in relation to objects, whereas the two control groups had similar ON and AN scores.^31^ Executive functions, attention, spatial and temporal orientation, memory, and language were also assessed in PD patients without dementia (n=50), and a group of healthy controls (n=42). AN was the only test to significantly discriminate the groups.^32^

A similar study with PD patients (n=32) sought to assess the ability of these patients on the ON and AN tests compared with healthy controls (n=15). The effect of a conceptual dimension (manipulability of the items) on ON and AN was also assessed. The noun-verb stimuli were re-categorized into manipulable and non-manipulable items (i.e. objects which can or cannot be manipulated and actions which do or do not involve fine hand movements). PD patients showed ON and AN deficits in comparison to controls, and these patients, but not controls, were significantly more impaired on AN than on ON. No effect of manipulability of objects or actions was observed on naming performance.^14^ The authors did not comment in the manuscript whether this group of PD patients presented dementia.

Forty-nine PD patients and 19 healthy seniors were assessed by an action naming test composed of two subsets of 25 verbs with different degrees of motor component. The aim was to support the hypothesis that, for the brain to process words with motion content, this processing would depend on neural activity in regions involved in motor planning and execution. The authors provided evidence supporting this hypothesis because PD patients performed worse on pictures with high motor content than on pictures with low motor content.^33^

One investigation on cognition in a group of patients with Friedrich Ataxia (n=36) and healthy controls (n=31) applied a large neuropsychological battery. The patients had poorer performance in different domains, but especially on executive functions. Patients also showed a significantly poorer performance in action naming.^34^

*Action Naming in stroke and lobectomies* - The studies investigating AN in stroke and lobectomy assessed different types of aphasia and sought to better understand the verbal processing in these patients mainly through linguistic theories, rather than through association with brain areas.

The conceptual factor "instrumentality" and the lexical factor "name noun-related" could play a role in anomic aphasic patients and were investigated in a study with anomic aphasic (n=19) and Broca's aphasic patients (n=15). In the anomic aphasic group, instrumental verbs were shown to be better preserved than non-instrumental verbs, but not among Broca's aphasic patients. Verbs whose name was related to a noun were better retrieved than verbs without a name relation, in all groups.^35^ In the view of the authors, the most interesting finding was that the anomic aphasic patients had better instrumental verb naming. The authors explained this finding through a mechanism supported by the spreading activation theory. Normally, anomic aphasics cannot activate the target lemma of verbs. However, the co-activation of the lemma of an instrument related to a verb helps reactivate the lemma of the verb.

The naming and dissociation of different types of verbs (transitive, intransitive, and ergative verbs) were verified in different types of aphasias. Fifty-eight mild-to-moderate Italian aphasic patients, and 45 normal controls participated in the study. The agrammatic aphasics showed a dissociation of types of verbs with impaired transitive verb naming. In nonfluent disorders, only noun superiority occurred. In fluent disorders, noun superiority in Wernicke's aphasia and verb superiority in anomic aphasia was found. In general, the nonfluent aphasia patients showed more ergative than intransitive verb impairment.^36^ The data did not only indicate a verb-noun dissociation, but also dissociation for type of knowledge underlying the corresponding lexical labels, both at the lemma level and the semantic level. The observed verb-noun dissociations could not be taken as a proof that verbs and nouns are stored in functionally and anatomically separate mental lexicons, according to the authors of this investigation. However, other findings also indicated that they differ for the type of knowledge underlying the corresponding lexical labels, both at the lemma level and at the semantic level.

The semantic attribute hypothesis posits that lexical access is mediated by brain systems that process the most definitive attributes of specific concepts. The competing grammatical role hypothesis posits that access to a word depends on the grammatical role it plays in a sentence. These two hypotheses were tested in experimental subjects who had undergone left anterior temporal lobectomy (LATL) (n=15) or right anterior temporal lobectomy (RATL) (n=15) by assessing confrontation naming of living things (nouns), tools/implements (nouns), non-human actions (verbs), and human actions (verbs). Patients with LATL were worse at naming tools/implements and human actions than RATL patients.^37^ This finding corroborates the semantic attribute hypothesis.

The main results from clinical studies employing AN are summarized in Table 3.

**Table 3:** Summary of main results in clinical studies with AN.

Studies	Clinical Groups Studied	What was analyzed?	Main results
Druks et al. (2006)	AD, HC	ON, AN	AD, HC: AN<ON
Gainotti et al. (2008)	MCI-HA, MCI-MSI	ON, AN	ON: MCI-MSI< MCI-HA
Hillis, Oh & Ken (2004); Ostberg et al. (2005)	nfPPA, FTD-ALS, fPPA	ON, AN	nfPPA and FTD-ALS: ON<ANfPPA: ON<AN
Cotelli et al. (2006)	bvFTD, nfPPA, SD, PSP, CBD, HC	ON, AN	bvFTD, nfPPA, PSP, CBD: AN<ONSD: ON<ANAN: nfPPA, PSP, CBD< bvFTD
Rodríguez-Ferreiro et al. (2009)	PD, AD, HC	ON, AN, effect of manipulability	PD: AN<ON
Rodríguez-Ferreiro et al. (2010)	PD, HC	ON, AN	AN: PD<HC
Cotelli et al. (2007)	PD, HC	ON, AN, effect of manipulability	AN, ON: PD<HCPD: AN<ON
Herrera et al. (2012)	PD, HC	AN, motor content	PD: high motor content<low motor content
Nieto et al. (2012)	FA, HC	AN	AN: FA<HC
Jonkers & Bastiaanse (2007)	Anomic Aphasics;Broca's Aphasics	AN, instrumentability, name noun-related	Anomic Aphasics: non instrumental verbs< instrumental verbsAll groups: verbs without name related to a noun<verbs with name related to a noun
Luzzatti et al. (2002)	Nonfluent aphasics, fluent aphasics, HC,	AN (transitive, intransitive, ergative verbs), ON	Nonfluent aphasia: AN<ONNonfluent aphasia: ergative verbs<intransitive verbsAgrammatic aphasia: Transitive verbs< intransitive and ergative verbsWernicke aphasia: AN<ONAnomic aphasia: ON<AN
Lu et al. (2002)	LATL, RATL	ON (living things, tools/implements), AN (non-human actions, human actions)	Naming tools/implements and human actions: LATL<RATL

AD: Alzheimer disease; AN: action naming; bvFTD: behavioral variant Frontotemporal Dementia; DLB: dementia with Lewy bodies; FA: Friedreich ataxia; HC: healthy control; LATL: left anterior temporal lobectomy; MCI-HA: Mild Cognitive Impairment due to hippocampal atrophy; MCI-MSI: Mild Cognitive Impairment due to multiple subcortical infarcts; nfPPA: nonfluent Primary Progressive Aphasia; ON: object naming; NPH: normal pressure hydrocephalus; PD: Parkinson disease; RATL: right anterior temporal lobectomy; SCI: Subjective Cognitive Impairment; PSP: progressive supranuclear palsy

**Neuroimaging studies with Action Fluency.** Neuroimaging studies may help toward understanding the neural mechanisms underlying AF task. Three such studies were found in the present search.

One of these studies aimed to compare the effectiveness of AF and of a Verb Generation Task (VGT) in presurgical evaluation of language in patients who were candidates for neurosurgery based on functional magnetic resonance imaging (fMRI). The VGT consisted of the generation of a verb that is semantically related to a presented noun (for example, "eat" for "spoon"). Eighteen controls and 58 patients that were candidates for neurosurgery (16 subjects with temporal lobe epilepsy and 42 with brain lesions), all right-handed, were recruited. In both samples, the VGT produced more specific activation of left Broca's area. In contrast, the AF yielded wider and more intense activation of the left Broca's area in controls, as well as other activations in the dorsolateral prefrontal cortex and the striatum. Additionally, both studies showed good agreement on language dominance derived from the tasks, although there was some variability in laterality index scores.^38^

The other investigation assessed AF using single-photon emission computed tomography (SPECT) in patients with varying degrees of cognitive decline (n=93), ranging from very mild subjective impairment to AD. No information on patients' handedness was given in the paper. Action Fluency impairment was associated with temporal lobe hypoperfusion and lower education while impaired fluency of nouns was associated with low perfusion of the parieto-temporo-occipital region and older age. The analysis of perfusion in the temporal region showed primary involvement of the temporal pole and the medial temporal lobe in AD. According to the authors, this may reflect the parahippocampal region pathology that appears early in neurodegenerative diseases.^39^

The most recent study evaluated semantic, verb and letter fluency in MCI and AD patients using novel techniques for measuring word similarity in fluency lists and a region of interest (ROI) analysis created with MRI data of gray matter correlates. The most important findings for this review were the correlations with the AF and gray matter. The study found correlations with left dorsal frontal and lower temporal ROIs, and with right lower temporal, mesial temporal, occipital, and inferior parietal/superior temporal ROIs. The right lower temporal ROI also remained significant in a regression model that included gray matter values from the left lower temporal ROI. This suggests that the finding on the right was not due merely to symmetry of atrophy (although the finding on the left might be). The authors suggested that the gray matter in the left dorsal frontal and right lower temporal ROIs may mediate the relationship between diagnosis and fluency for verbs.^40^

The main results for neuroimaging studies and AF are summarized in Table 4.

**Table 4:** Summary of the main results in neuroimaging studies with AF.

Studies	Clinical Groups Studied	Technique utilized	Brain areas related with AF
Sanjuán et al. (2010)	HC, temporal lobe epilepsy, brain lesions	fMRI	Left Broca's area, dorsolateral prefrontal cortex, striatum
Ostberg et al. (2007)	AD, MCI, SCI	SPECT	Temporal lobe hypoperfusion, temporal pole and medial temporal lobe in AD
Clark et al. (2013)	MCI, AD	ROI (MRI)	Left dorsal frontal area; right lower temporal area

AD: Alzheimer disease; HC: healthy control; MCI: Mild Cognitive Impairment; MRI: magnetic resonance imaging; ROI: region of interest; SCI=Subjective Cognitive Impairment; SPECT: single-photon emission computed tomography.

**Neuroimaging studies with Action Naming.** Four studies on neuroimaging and AN were retrieved.

The study of Piras and Marangolo^41^ analyzed aphasic patients with left hemisphere lesions (n=16) using voxel-based lesion symptom mapping (VLSM), and showed an association between naming of nouns and regions of the left superior temporal lobe. They also found an association between verb naming and a large region extending from the prefrontal area to areas of the left superior temporal lobe. This finding was confirmed by the same authors in another study in which 20 left hemisphere stroke patients named four types of images: nouns, verbs, and noun-noun (NN) and verb-noun (VN) compounds. The performances were analyzed using VLSM. The results showed that while NN compounds involved the same temporal areas of nouns, verbs and VN compounds involved different fronto-temporal regions (although belonging to the noun category).^42^

The aim of the third investigation in this category was to test the hypothesis that concept formation deficits associated with an extramotor neurocognitive network involving executive and semantic resources could be found in some ALS patients. Forty-one patients with clinically definite ALS were assessed with the Delis-Kaplan Executive Function System (D-KEFS, Free Sort Test and the Recognition Sort Test Condition) along with neuropsychological tests measuring naming, semantic memory, and executive control. High-resolution T1 structural Magnetic Resonance Imaging (MRI) was used to examine cortical thickness in a subset of 16 ALS patients. Some patients with ALS produced comparatively low scores on measures of free and recognition sorting. Reduced sorting performance was related to lower performance on tests assessing both AN and word meaning. Finally, regression analysis related lower recognition sorting performance to greater cortical thinning in prefrontal and parietal cortex. This finding may reflect the vulnerability of an action/semantic network in ALS.^43^

Performance on comprehension and production of nouns and verbs was assessed in patients with PSP (n=10) matched with healthy controls (n=10). The PSP patients also underwent SPECT measurements. The PSP patients had poorer performance on verbs as compared to nouns on various lexical-semantic tasks (oral and written confrontation naming, auditory and visual single-word comprehension) and were worse than healthy controls. All PSP patients showed significant hypoperfusion in the inferior frontal gyrus (IFG) on SPECT. The authors hypothesized that impairments in production and comprehension of verbs in PSP patients could be secondary to semantic deficits affecting the conceptual category of actions and that neural systems in posterior frontal cortical areas (mainly involving the IFG) may be critical for processing the conceptual category of actions.^44^

**Whole-head magnetoencephalography studies with Action Naming.** Only two studies using whole-head magnetoencephalography (MEG) were found, both of which used the AN. No studies using MEG and AF were found.

In one of the studies, estimates of activations of the lateral occipital, inferior parietal, superior temporal, basal temporal and inferior frontal cortical areas were carried out in healthy right-handed subjects (n=8). The normal magnetic flux to the scalp surface was recorded with MEG while subjects silently generated verbs in response to visual stimuli. The peak latencies in the studied regions progressed from the posterior to anterior regions. The earliest peak latency was in the lateral occipital cortex bilaterally, while the latest was in the inferior frontal gyrus (IFG) pars opercularis, which is part of classical Broca's area, and also IFG pars triangularis The index of hemispheric asymmetry was significant in the IFG pars opercularis and stronger for the left hemisphere.^45^

The second study recorded cortical dynamics with MEG in 10 healthy subjects during AN and ON tasks.^46^ The activation sequences in AN and ON were similar, showing a consistent advance from the occipital to posterior temporoparietal and further to the left frontal cortex, without consistent involvement of the classical left inferior frontal (Broca) and temporal (Wernicke) language areas.

The main results for neuroimaging and whole-head magnetoencephalography studies with AN are summarized in Table 5.

**Table 5:** Summary of the main results in neuroimaging and whole-head magnetoencephalography studies with AN.

Studies	Clinical Groups Studied	Technique utilized	Brain areas related with AN
Piras & Marangolo (2007)	Aphasia	VLSM	left prefrontal area to left superior temporal area
Piras & Marangolo (2010)	Stroke	VLSM	Verbs and verb-nouns: different fronto-temporal regions
Libon et al. (2012)	ALS	MRI (cortical thickness)	prefrontal and parietal cortex
Daniele et al. (2013)	PSP, HC	SPECT	hypoperfusion inferior frontal gyrus
Breier & Papanicolaou (2008)	Healthy subjects	MEG	First activation: lateral occipital cortex bilaterallyLatest activation: pars opercularis, triangular part of inferior frontal gyrus (stronger in left hemisphere)
Soros et al. (2003)	Healthy subjects	MEG	Occipital to posterior temporoparietal and further to the left frontal cortex

AN: action naming; ALS: amyotrophic lateral sclerosis; HC: healthy control; MEG: magnetoencephalography; MRI: magnetic resonance imaging; PSP: progressive supranuclear palsy; SPECT: single-photon emission computed tomography; VLSM: voxel-based lesion symptom mapping.

## DISCUSSION

The aim of the present review was to analyze the information available in the scientific literature on the mechanisms (brain processes) and clinical applications of the AF and AN tasks. The most accepted mechanism for AF is primarily dependence on the following sequence: communicative intentionality (planning and communicative initiative), semantic category access, lexical access, phonological programming and, finally, planning and organization of the articulatory motor act. These processes suggested that initially, an activation of the dorsolateral frontal cortex occurs, because this region encompasses storage of this information or is related to the integration of the action-related semantic content. Subsequently, the prefrontal cortex is recruited for the word generation.

Based on the reviewed neuroimaging, clinical and neurophysiological studies, it is possible to understand some of the brain mechanisms underlying AF. Production of verbs in AF occurs with the recruitment of frontal areas and frontal brain circuits, such as Broca's area, the dorsolateral prefrontal cortex, dorsal frontal cortex, and the striatum, with a lateralization to the left hemisphere in right-handed patients.^38,40^ However, this predominance of frontal activations does not mean that other regions are not necessary. The study of patients with cognitive impairment (MCI or AD) showed temporal hypoperfusion associated with impaired AF.^39^ According to these findings, AF depends on frontal and temporal circuits also involving subcortical regions of the basal ganglia. These neuroimaging findings corroborate clinical findings that showed impaired AF in patients with involvement predominantly in frontal circuits as in FTDs,^21^ but also in those with frontostriatal impairment as in PD^7,22^ and as well as temporal in AD and MCI.^9^

Action Fluency can be more dependent on frontal cortical and fronto-striatal regions and even cerebellar regions since impairments in AF have been observed in patients with impairments of these areas, such as in PD, schizophrenia, HIV-1 infection and Friedreich's ataxia, compared to individuals with temporal impairments.^19,22-27^ This also indicates a certain relationship between the pathways for access to the semantic knowledge of the action and brain areas related to the motor action itself.

Regarding the neural mechanism underlying the AN task, we speculate that it involves several cognitive processes such as perception and visual recognition, recall of semantic memory, lexical access, phonological programming and finally, the execution of the articulatory motor plan. Therefore, to name a figure, we hypothesize that it is necessary: first, to activate regions of the occipital cortex for the perception and recognition of the visual image; second, to activate parietal regions for spatial and temporal perception of the action depicted in the picture; third, to activate different temporal areas related to memory and that are responsible for the retrieval of the concepts involved in the integration of image and semantic content required to access the lexical representation of the concept involved, concomitantly with the activation of frontal areas related to motor representation of the action also involved in the representation of linguistic action; lastly, to activate the primary motor cortex for phonological programming and Broca's area for the production of the articulatory motor act.

Most of the studies, whether clinical or neuroimaging, have used ON and AN strategies to understand the underlying mechanism of the grammatical dissociation. The differences between the naming of nouns and verbs seem to lie in the stage of evocation of semantic and lexical concepts. ON has been shown to be associated with regions of the left superior temporal cortex, while AN is linked with a large region extending from the prefrontal cortex to the left superior temporal areas,^41^ with some involvement of the left parietal^43^ cortex. The findings of this review converge to a theory in which the dissociation between AN and ON does not occur solely because of grammatical factors.^33,36,37,42^ Thus, we are led to think that the neural mechanisms for the AN could be related to mechanisms of action semantic knowledge rather than grammatical knowledge of a specific word class. In addition to the frontal regions activated during AN, it is probable that other areas are previously activated according to the following sequence: lateral occipital cortical area, inferior parietal, superior temporal, inferior frontal and basal temporal.^45^ However, there is evidence that the frontostriatal circuit can carry out this function, in view of the findings with PD.^14,31,32^ There is also evidence that subcortical regions which can be related to the representation of action are involved in this task.^11^

Finally, it is important to discuss the applications of AF and AN. A role in the differential diagnosis of PPA has been suggested for both AF and AN, since there are specific patterns of language impairment,^8,21,30^ but there is still a need for studies involving larger samples and more appropriate analyses.

The AF might represent a new measurement of language and executive function. Studies of construct validity of AF have shown a correlation with other tests that assess frontal functions, especially those assessing executive functions (planning, processing speed, and cognitive flexibility). The fact that the correlations are modest may suggest that this task provides unique measures of executive function itself.^12,15^ As verbs are more grammatically complex words than nouns, tasks that use verbs tend to be more influenced by schooling and more sensitive to brain impairment (MCI and AD) and to cognitive changes related to aging.^9,10,16,19^

Some studies have used different types of analyses where these can be explored in future studies, for example, the analysis of specific characteristics of verbs such as manipulability (using the hands in action),^14^ instrumentality,^35^ verbal predication,^36^ and analysis of error types.^13^ These possibilities of analysis may further our understanding of cognitive processes and language, as well as the processes involved in certain diseases.
